# Underlying Immune Mechanisms Involved in Cow’s Milk-Induced Hypersensitivity Reactions Manifesting as Atopic Dermatitis

**DOI:** 10.7759/cureus.27604

**Published:** 2022-08-02

**Authors:** Derek S Weimer, Michelle Demory Beckler

**Affiliations:** 1 Biomedical Sciences, Nova Southeastern University, Dr. Kiran C. Patel College of Allopathic Medicine, Fort Lauderdale, USA; 2 Microbiology and Immunology, Nova Southeastern University, Dr. Kiran C. Patel College of Allopathic Medicine, Fort Lauderdale, USA

**Keywords:** cow’s milk protein allergy, food and nutrition, skin health, skin, immunology, hypersensitivity reactions, milk allergy, atopic dermatitis, dermatology, allergy

## Abstract

Of the many symptoms associated with cow’s milk allergy (CMA), many populations face the burden of the appearance or worsening of atopic dermatitis (AD) when consuming milk products. Due to the prevalence and possible severity of symptoms, it is important to understand the underlying immune mechanisms involved in such reactions. Hypersensitivity reactions are exaggerated immune responses to often benign antigens, many times resulting in a cascade of pro-inflammatory processes. Of the four major types, type I and IV are of most relevance when considering atopic dermatitis worsened by cow’s milk. Considered a “true allergy,” type I (immediate) hypersensitivity reactions occur within hours after secondary exposure to an allergen and are primarily driven by antibodies and humoral immune responses. On the contrary, type IV (delayed) hypersensitivity reactions are driven by cell-mediated responses involving T-cell activation. Due to the array of symptoms induced by these complex reactions, it is imperative to diagnose early and treat appropriately. In this literature review, we aim to highlight the primary underlying immune contributors to hypersensitivity reactions, discuss AD as a manifestation of hypersensitivity reactions to cow’s milk, and consider current and future treatment options for combatting hypersensitivities manifesting as AD.

## Introduction and background

The United States (US) Food and Drug Administration (FDA) reports milk among the top eight food allergens affecting the US population [1]. Moreover, the US Department of Agriculture recommends adults consume three cups per day and children consume 2-2.5 cups per day [2]. Despite these recommendations, the prevalence of milk allergy remains stable throughout many studies and across many years [3-5]. More recently, it was reported that milk allergy (i.e., IgE-mediated) affects nearly 4.7 million US adults [3], with the most affected age group being 18-29 years of age, which is approximately 2.4% (95% CI: 2-2.9) [3]. Among individuals with a cow’s milk allergy (CMA), 39.3% (95% CI: 35.2-43.5) report having a severe reaction to milk within their lifetime, and 47% (95% CI: 42.8-51.1) report a milk allergy-associated hospitalization within their lifetime [3]. Given the prevalence and possible severity of these reactions, it is important to understand the underlying immune mechanisms, inducers, and mediators of these hypersensitivity reactions.

Approximately 5% of milk composition contains carbohydrates primarily lactose, glucose, and galactose, while about 2.9%-3.5% of milk contains various proteins, among other components, including vitamins, minerals, nucleotides, immunoglobulins, and others to a lesser degree [6]. According to MyPlate, the major benefits of daily milk consumption include improved bone structure and growth due to calcium and, if fortified, vitamin D content [2]. Multiple studies have shown similar benefits of human milk consumption when compared to bovine milk, including increased antibody production in infants, intestinal barrier protection, and improved overall diversity within the gut microbiota [7]. Despite these and other benefits, cow’s milk and/or its components can induce severe hypersensitivity reactions in certain patient populations.

Two important contributors to hypersensitivity manifestations in patients with CMA are whey and casein proteins [8,9]. Poulsen et al. reported that despite pasteurization (heating the milk to 720°C, followed by intense cooling for preventing bacterial growth of various bacteria and other harmful microorganisms) and homogenization (processing milk under high pressure to disperse fat molecules producing consistency and orderly texture), milk still induced anaphylactic shock within milk-sensitized mice, whereas no reactions were exhibited to raw, unprocessed milk [10]. This finding suggests that these two crucial milk-producing processes have little to no effect on hypersensitivity prevention. Casein proteins in milk are contained within large fat micelles consisting of a 𝜅-casein core [10]. These micelles protect the 𝛼- and 𝛽-casein proteins, thus preventing them from being exposed within the milk. When homogenized, the fat micelles are disrupted and thus break apart into smaller fat molecules that expose the casein proteins and, to a lesser extent, the whey proteins. Eventually, once settled, the milk forms a structured protein layer amid the fat layers within the milk [10]. With increased exposure to casein and, to a lesser extent, whey proteins, there becomes an increased likelihood of exposure within the body, potentially leading to increased immunological responses in those with cow’s milk hypersensitivities [10].

Hypersensitivity reactions are exaggerated immune responses to often benign antigens, many times resulting in a cascade of pro-inflammatory processes. Type I, II, and III hypersensitivity reactions are mediated by antibodies and humoral immune responses. Type IV or delayed-type hypersensitivity reactions are considered cell-mediated and are directed by T-cells. The initiation of these hypersensitivities depends on the individual and the antigen. For example, many cow’s milk hypersensitivity reactions result from exposure via inhalation, contact, or ingestion of the antigen and ultimately lead to varying symptoms such as atopic dermatitis (AD), rashes, gastrointestinal bloating, diarrhea, and urticaria [9]. Of the four major types of hypersensitivity reactions first termed by Gell and Coombs in 1963, type I and type IV are of most relevance when considering atopic dermatitis worsened by cow’s milk [9,11].

Atopic dermatitis (AD) is an inflammatory skin disorder characterized by eczematous lesions, intense pruritus (i.e., itching), and a chronic or relapsing disease course [12]. In the United States, eczema is considered the most common chronic inflammatory skin disease, affecting nearly 31.6 million people (10.1%) [13]. Once considered to be a childhood disease, recent studies have shown otherwise [14]. Of the 13,000 individuals enrolled in the longitudinal birth cohort studies examined, no statistically significant differences in AD prevalence were observed from childhood to adulthood [14]. This indicates disease progression from childhood to adulthood, although intermittent remission commonly seen with eczema was not measured in detail. Symptoms of eczema are considered heterogeneous in nature due to the vast differences appreciated within children and adult populations, although some underlying themes do exist. In infant populations, there are often symptoms throughout the face, cheeks, and trunk, excluding the “nappy area” (i.e., diaper rash region). Infant symptomology can include acute lesions characterized by poorly defined erythema (i.e., redness of the skin), vesicles, excoriations, and/or serous exudates [12]. In children two years and older, AD is increasingly chronic and characterized by symptoms of xerosis (i.e., dry skin), palmar erythema, and poorly defined lesions commonly found within areas where a joint folds (i.e., flexor surfaces) and accompanied by skin thickening (i.e., lichenification) [12,15]. Lastly, affected adults commonly present with diffuse eczema, while localization is seen to a lesser extent. The commonly affected areas include the hands, shoulders, and scalp [12]. As a systemic disease, AD is quite challenging when considering treatment options. The cause of AD is multifactorial (e.g., genetic, environment, and diet), increasing the complexity of the disease [12]. With an array of symptoms driven by numerous immune regulators and its multifactorial nature obscuring its etiology, AD is difficult to diagnose and treat, leaving many with untreated disease and overall lower quality of life.

Despite extensive research focusing on the influence of cow’s milk hypersensitivity on various gastrointestinal symptoms, there are other manifestations of cow’s milk hypersensitivities, including those resembling atopic dermatitis. However, the abundance of studies on dermatological manifestations of cow’s milk hypersensitivities is much less than that of the gastrointestinal tract. In this literature review, we aim to highlight the primary underlying immune contributors to hypersensitivity reactions, discuss AD as a manifestation of hypersensitivity reactions to cow’s milk, and consider current and future treatment options for combatting hypersensitivities manifesting as AD.

## Review

Type I hypersensitivity reactions and atopic dermatitis

Type I hypersensitivity reaction, also considered a true allergy, is similar to any other hypersensitivity reaction in the body in that the main goal is to protect host (i.e., self) vital tissues from potentially harmful foreign invaders (e.g., bacteria and parasites). With allergies (i.e., type I hypersensitivities), immune-activated inflammatory reactions result from benign objects (e.g., peanuts, milk, and soy) that are viewed as harmful by the body, called allergens. Type I hypersensitivity reactions can occur within minutes of secondary exposure to an immune-inducing antigen. Following primary exposure to an allergen, no manifestations typically result. Once initiated by the T-helper (TH) type 2 cells and additional mediators, isotype switching within B-cells occurs, thus producing IgE antibodies at a rapid level despite a lack of clinical symptoms, a process often called “sensitization” [11]. On re-exposure, however, classical allergy-related symptoms become evident after the occurrence of two crucial phases.

The early phase includes the allergen cross-linking of IgE antibodies with mast cells and basophils [11]. Within minutes after exposure to the allergen, these cells release their granules (i.e., degranulate) rapidly, releasing histamine, proteases (e.g., tryptase and chymase), lysosomal enzymes, and other mediators from their centers [11]. This creates a disordered microenvironment in an attempt to eliminate the unrecognizable foreign molecule from the body.

Occurring within four to eight hours after exposure, the late phase involves the initiation of various cytokines such as interleukins (ILs) (e.g., IL-1, IL-4, IL-5, and IL-13), tissue necrosis factor (TNF), and granulocyte-monocyte colony-stimulating factor (GM-CSF) produced by mast cells [11]. This release in combination with an array of other contributing factors induces common allergy associations, including respiratory symptoms such as rhinorrhea and wheezing and, in more serious reactions, anaphylaxis [8,11]. In addition to these, IgE-mediated reactions are appreciated on the skin as hives and angioedema [8].

Type I hypersensitivity reactions are characterized by their immediate inflammatory nature after being faced with an immune-inducing antigen. When considering AD, a chronic inflammatory skin disease, it calls to consider the possible interplay between AD and CMA pathogenesis [16]. When considering pathophysiology, patients with AD show common histological findings, the most prevalent being a dysregulated epidermal barrier. This is thought to be chiefly attributed to the hyperactivation of T_H_2 (T-helper 2) cells [12,16]. Being primed by various ILs in the microenvironment (e.g., IL-4, IL-5, and IL-13), T_H_2 cells are tightly associated with type I hypersensitivity reactions due to their role in releasing their own IL-4, which can drive isotype switching in B-cells and the subsequent rapid production of IgE antibodies. It has been shown in cell cultures that on the reintroduction of cow’s milk after elimination, those cells with CMA exhibited increased spontaneous IL-4 production as compared with those without CMA [17]. This finding is suggestive of a central role for IL-4 in atopic dermatitis and, more specifically, in IgE-mediated allergies [17]. Additionally, T_H_2 cells release IL-5, which induces eosinophil degranulation and prepares mast cells for future allergen interactions [11]. By examining polymorphisms within T_H_2-related genes in patients with AD, IL-4 and IL-13 have been shown to contribute most to epidermal barrier dysfunction, promoting the characteristic dry skin [16]. Dry skin consists of suboptimal skin layers that have been demonstrated to increase sensitization to many other benign objects (e.g., milk and peanuts) through decreased barrier protection. This can contribute to a worsening of food allergies and future allergic episodes [16]. Exhibiting a somewhat cyclic fashion, this sensitization can eventually promote the migration of inflammatory cells into many AD lesions, leading to the continuation of worsening skin conditions [16].

The underlying interplay between CMA and AD in infant and children populations has been widely researched for many years. In 1996, Baehler et al. demonstrated, through a double-blind placebo-controlled food challenge (DBPCFC) method, the distinct differences between type I and type IV hypersensitivity reactions in infants and young children with CMA [23]. The oral food challenge (OFC) test, specifically the double-blind placebo-controlled food challenge (DBPCFC) test, is the gold standard for diagnosing a food allergy [18-22]. By conducting a DBPCFC test, the study found that dermatological symptoms of urticaria, erythematous rash, and angioedema were noted primarily in the immediate hypersensitivity reaction group (62.5% of participants with CMA), whereas gastrointestinal symptoms were noted in the delayed-type hypersensitivity group (28.8% of participants with CMA) [23]. However, more recent studies have shown dermatological manifestations occurring within both mechanisms [24]. Interestingly, type I-mediated reactions were found to occur more frequently within infant and children populations. In a large study examining IgE levels and severity of AD in children, a positive correlation was found between increased IgE levels and increased severity of disease, indicated by a higher SCORing Atopic Dermatitis (SCORAD) score (a universal system implemented in determining the severity of AD) [25]. This study demonstrates the important role of IgE-mediated reactions in AD pathogenesis and severity. Moreover, after the reintroduction of milk to children with a previous four-month dairy elimination diet, the highest IgE humoral responses (i.e., type I) against 𝛼-lactalbumin, 𝛽-lactoglobulin, and casein were noted in 59.3% of patients with AD and CMA [26]. These findings suggest that the studied cohort was likely exhibiting a “true allergy” derived from a type I hypersensitivity mechanism. Interestingly, as part of the study of Wahn et al., the number of children sensitized to milk allergy did not increase during the second year of life [25]. This provides an indication of cow’s milk sensitization occurring primarily within the first year of life; however, this fails to consider adult-onset disease.

After previously being considered childhood diseases that most outgrow, adult populations affected by AD and CMA remain steady [3,14]. Despite a large prevalence, to date, we have only identified one randomized controlled trial (RCT) studying AD and concomitant CMA in adolescent and adult populations. Of the limited research, Celakovska et al. conducted a study examining this very concept [22]. Of the 179 participants enrolled in the study (51 males and 128 females), only one participant received a positive result from a double-blind placebo-controlled lyophilized (i.e., freeze-dried) food challenge, accompanied by worsening AD [22]. The case was a 48-year-old male who suffered from AD for seven years prior to the study (i.e., adult onset) and experienced almost permanent eczematous lesions for two years prior to the start of the study. SCORAD was the tool used to clinically determine the severity of AD, in which he was evaluated at 53.5, indicating severe AD, prior to any diet change. On the elimination of cow’s milk from his diet, SCORAD levels decreased at the 3-, 6-, 9-, and 12-month follow-up visits from 28.2 to 25, to 24, to 20. The SCORAD value of 20 at the 12-month follow-up was statistically significant when compared to before the diet change [22]. Although demonstrating AD and CMA in a type I occurrence characterized by immediate worsening of AD symptoms, this study is vastly limited in scope. This is primarily due to the small sample size and stringent guidelines for food allergy testing, while other studies have noted simply the DBPCFC test to be the gold standard for diagnosing CMA [18,21]. When considering adults with AD and CMA, a lack of research hinders the treatment and identifiable commonalities as noted in infants and children.

As outlined, AD and CMA have common underlying immune mechanisms for the generation and worsening of dermatological symptoms seen in the infant, children, and adult populations. However, hindered by small sample sizes, a complex disease course, and varying symptomatology, more research is warranted.

Type IV hypersensitivity reactions and atopic dermatitis

Type IV (i.e., delayed-type, non-IgE-mediated) hypersensitivity reactions are characterized by their T-cell activation and onset time of symptoms (i.e., 48-72 hours after exposure) [8]. On initial exposure to the allergen, primed T-cells are activated, leading to a cascade of various events depending on the type of T-cell that is activated (e.g., CD4+ or CD8+). Acting as a snowball effect, various inflammatory-associated cells, such as macrophages, neutrophils, and eosinophils, become peripherally activated as well, eventually leading to an overall inflamed microenvironment with the potential to injure surrounding tissues and elicit accompanying symptoms such as swelling and hives [11]. Dermatological symptoms are similar when compared to type I reactions. However, the prevalence of type IV reactions is typically lower than those of type I. Although varying between studies, prevalence tends to be below 40% of participants, with some even being below 25% [8,26-28]. Due to the prolongation of symptom onset, sometimes days after initial exposure, the diagnostic ability becomes increasingly difficult [8,9].

Although not considered a true allergy as type I hypersensitivities, type IV reactions do contain similar underlying immune characteristics. Contradictory to type I hypersensitivities, these mechanisms are directed by T-cells. There are four subtypes: IVa, IVb, IVc, and IVd [11]. When considering cow’s milk hypersensitivity and AD, type IVa is of most relevance, although all potentially play a role in the generation and maintenance of symptoms [11,18,26,28].

Type IVa is described as the classical definition of a type IV hypersensitivity. Following exposure to the allergen, an antigen-presenting cell (APC) (e.g., Langerhans cells (dendritic cells) and macrophages) is stimulated and transported to the appropriate lymph node where it binds to a naive CD4+ T-cell using two separate methods (MHC II binding and CD28 to B7 binding). This binding results in the release of IL-12 from the APC and the subsequent activation of the naive helper cell to a T_H_1 (i.e., T-helper 1) cell [11]. As a T_H_1 effector cell, IL-2 and interferon gamma (IFN-𝛾) are released rapidly, thus eliciting a cascade of pro-inflammatory events. One important event that occurs is the activation of macrophages, leading to increased effector molecules IL-1, IL-6, and TNF-𝛼 [11,26]. The release of these pro-inflammatory molecules induces symptoms of swelling (i.e., edema), gastric discomfort, and hives, among other symptoms [11].

Testing peripheral blood mononuclear cells (PBMCs) to various cow milk components (i.e., 𝛼-lactalbumin, 𝛽-lactalbumin, and casein) after a four-month elimination diet, Motrich et al., as detailed above, also examined type IVa involvement in patients with known CMA and AD symptoms [26]. The introduction of a cow’s milk antigen mixture containing the various milk components (𝛼-lactalbumin, 𝛽-lactoglobulin, and casein) led to significantly higher levels of TNF-𝛼 concentrations in the cell cultures of 40.7% of patients with AD exhibiting allergic-like symptoms [26]. This finding suggests that these patients exhibited specifically type IVa hypersensitivity responses. Unfortunately, with a small sample size, an age range of one to seven years of age, and limited details discussing the chronological onset of symptoms, the study is limited in scope.

Contrary to the pro-inflammatory factors mentioned above, Sütas et al. identified IL-10, an anti-inflammatory cytokine, as a possible integral component of delayed-type hypersensitivity reactions in infants with CMA and AD [28]. In this study, 76 DBPCFC tests across 56 infant participants with a mean age of 15 months showed that late-reacting patients were characterized by positive skin patch tests and negative skin prick test (SPT) results along with lower IL-10 concentrations prior to the challenge, indicating a higher inflammatory status. Atopy patch tests are primarily employed when considering patients with symptoms indicative of a delayed or non-IgE-mediated mechanism. Unfortunately, these tests have variable sensitivity and specificity, suggesting that they are nondiagnostic when used alone [16,18]. The other modality used within the study includes skin prick tests (SPTs). These are noninvasive and are generally a quick method for detecting sensitivity. Despite the simplistic advantages, SPTs have low specificity, and the methods are not standardized, suggesting that this test is also nondiagnostic when solely used [18]. Patients with late-onset reactions (29/51; 57%) showed an average cumulative onset time of symptoms of 54 hours after the last dose eliciting the reaction [28]. With a lower overall IL-10 level prior to the oral food challenges, those with AD and CMA appeared with increased inflammation compared to those with tolerance, as expected. The median serum IL-10 concentration before the DBPCFC test in late reactions was 1.9 (0-4) pg/mL for 29 participants and was significantly higher in 12 participants with immediate onset reactions of 4.4 (2.6-5.2) pg/mL (P=0.007) [28]. Interestingly, after exposure to milk, those with an “allergy” were shown to spike in IL-10, surpassing those without suspected allergy. This is thought to contribute to the variability seen in skin tests with those of type IV reactions and reiterates the idea that IL-10 is released due to a reactive state against pro-inflammatory cytokines [28]. In addition, TNF-𝛼 and IFN-𝛾 were also measured using concanavalin A-stimulated cultures [28]. When comparing the immune effector and mediator, it was shown that those with delayed hypersensitivities reflected higher TNF-𝛼 and IFN-𝛾 concentrations (144.8 (46.1-270.4) and 7.6 (1-17.7) pg/mL, respectively) compared to those who exhibited tolerance (85.2 (42.1-130.4) and 6.5 (0.6-14.9) pg/mL, respectively) [28]. This finding is suggestive of the pronounced differentiations between the underlying immune mechanisms in patients with concomitant cow’s milk sensitivity and AD.

It is important to note that studies examining type IV mechanisms in these specific cohorts are extremely limited in the number of actual studies conducted, in addition to being limited in their small sample sizes and focus on infant and children populations. To our knowledge, no similar immunological studies have been conducted on adolescent and adult populations.

Treatments

AD presents with a multitude of different symptomological findings within the infant, children, and adult populations. Additionally, facing the added implications brought forth by cow’s milk allergy (e.g., vomiting, diarrhea, failure to thrive in children populations, and even anaphylaxis), it is pertinent to treat as early and safely as possible [10,16]. Five major treatment strategies have been found to be beneficial when treating AD associated with hypersensitivity reactions.

A diet in an attempt to decrease allergen exposure is known as an “elimination diet” [29]. In CMA, one might consider an elimination diet centering around the avoidance of cow’s milk and all associated products (e.g., specific bread, candies, and butter). Although a seemingly simple solution, when considering infant and children populations, it is crucial to understand the nutritional risks involved with implementing a strict diet eliminating all dairy. A cross-sectional study conducted by Boaventura et al. outlined the importance of cow’s milk on the overall nutritional status of preschool-aged children. It was found that preschoolers with dairy-modified diets initiated due to CMA exhibited slower growth rates and were considered at additional risk for obtaining proper nutritional status [30]. Additionally, the experimental group showed a significantly lower height (P=0.0043), decreased calcium intake (P=0.0033), and decreased lipid intake (P=0.0123) when compared to the control group [30]. With the many benefits associated with cow’s milk consumption in youth populations, it is crucial to first consider the risks and benefits when determining if a dairy elimination diet is the most appropriate solution. Due to the potential risk, accurate diagnosis becomes critical to avoid unnecessary restrictive diets. Dambacher et al. demonstrated that when diagnosing CMA through DBPCFC (compared to other tests), parents are more likely to be convinced that the symptoms are not associated with the cow’s milk protein (i.e., if negative test) and thus more likely to discontinue an unnecessary elimination diet in the long term [31]. This longitudinal study also stresses the importance of ensuring consideration for late reactions to CMA. Ultimately, proper diagnosis is crucial when deciding if a dairy elimination diet is the absolute best treatment for the patient when considering all variables.

Since first being introduced in the 1950s, topical corticosteroids have become the first-line defense in combating AD due to their vast functionality and high safety profile in topical use [32]. Corticosteroids carry the ability to target an array of inflammatory cell types and cytokines, including neutrophils, monocytes, lymphocytes, Langerhans cells, interleukins (e.g., IL-1𝛼, IL-1𝛽, and IL-2), and tissue necrosis factors (TNFs), among others, with high specificity. By inhibiting these molecules, corticosteroid lowers the ability to elicit symptoms, leading to improvements within the skin microbiome [32]. Topical corticosteroids are used in moderate to severe cases of atopic dermatitis and come in many preparations depending on the disease severity. In infants and adults with mild symptoms, a low-potency agent is indicated [33]. In children and adults with more severe symptoms, a mid-potency treatment may be beneficial, but its use is limited to short durations, although long-term use has been implemented when applied to lower-risk locations (e.g., trunk and extremities) [33]. High- and ultrahigh-potency topical corticosteroids do exist but are strictly used as a short-term treatment of lichenified areas in adult patient populations [33]. Although these medications are easy to use and have a wide range of functions, topical corticosteroids can induce varying topical and systemic adverse reactions. Notable topical side effects include localized skin reactions such as atrophy or thinning of the skin, contact dermatitis, and rosacea, among others [33]. Systemic adverse reactions include possible suppression of the hypothalamic-pituitary axis (HPA), growth retardation in children, cataract formation, and glaucoma development, although these are primarily noted in patients misusing the medication [33]. Although shown to be effective in patients with AD, we have not identified studies determining its effectiveness in patients with concomitant AD and CMA, although, with the studied mechanisms of action, similar effectiveness is hypothesized in the aforementioned populations.

Phototherapy as a therapeutic approach uses narrowband ultraviolet B (UVB) light to improve AD symptoms. In a large observational study, phototherapy significantly decreased patient SCORAD scores by an average of 18.9 units (P<0.001) and significantly improved the quality of life of the patients (P<0.001, measured using the Dermatology Life Quality Index) [34]. Some limitations to this therapy include cost, accessibility, and associated risk of photodamage and carcinogenesis [12].

As more research is produced, further understanding of the immunological mechanisms underlying the prevalent symptoms associated with AD has arisen. Monoclonal antibody treatment is now becoming increasingly common. Dupilumab, for example, is a human monoclonal antibody against IL-4R𝛼. Antagonizing this pro-inflammatory mediator produces a trickle-down effect, resulting in the double inhibition of IL-4 and IL-13 signaling, two major contributors to AD pathogenesis [12,35]. In the United States, this is the first biological therapy approved for moderate to severe AD in patients ages six years and older [12]. Renert-Yuval et al. explained that in addition to exhibiting great decreases in these T_H_2-related markers, there is also evidence of decreases in T_H_17-/T_H_22-related markers as well, indicating use beyond initial purpose [36]. Although exhibiting great potential, efficacy remains around 35%-40% of patients treated with dupilumab [36]. This reiterates the necessity for additional treatment options in addition to continued research into these novel biological therapies (i.e., biologics). More recently, studies have suggested introducing oral immunotherapy while on biologics in an attempt to improve efficacy. However, with increased safety concerns and mixed preliminary results, sufficient scientific evidence is lacking at this time, thus limiting its clinical application [37-39].

Although commonly prescribed for allergic reactions, the American Association of Dermatology (AAD) recommends sedating antihistamines as a short-term solution for insomnia associated with AD pruritus but denies any significant benefit in improving topical symptoms [40]. Data from the National American Medical Care Survey (NAMCS) found that from the years 2003-2012, there was an average of 27.7% of antihistamine prescriptions for the treatment of AD [40]. The top specialties included family/general practice (26.1%), internal medicine (33.1%), and other specialties not specifically noted (44.4%), while the lowest included dermatology (22.2%) and pediatrics (15.8%) [40]. This indicates that despite the AAD’s recommendations for monotherapy antihistamine use in treating AD, both sedating and nonsedating antihistamines are still commonly used in treating patients with AD. The limitation in decreasing dermatological symptoms is, in part, contributed to the minimal role histamine plays in the pathogenesis of AD. However, to our knowledge, no studies examine the relationship when including related CMA. Therefore, despite being the second-most commonly used medication for AD-associated pruritus, there exists insufficient evidence of significant benefit in patients with AD [40]. To address this, novel histamine 4 receptor (H4R) antihistamines have become an interest in treating AD. With one formulation currently in phase II clinical trials, H4R antihistamines, rather than the H1R antihistamines commonly prescribed for allergies, are currently being investigated due to H4R’s influence on the T_H_2 and T_H_17 inflammatory processes [36]. Preliminary studies have shown improvements, but no significant results have been documented, and thus, further research is warranted [36].

Discussion

Composed of high-quality proteins and a multitude of vitamins and bioactive molecules, it seems no surprise that cow’s milk remains a critical component of the American diet [2,6]. Despite the benefits, many populations face the burden of cow’s milk allergy (CMA). Within the United States, it is estimated that 1.9% (95% CI: 1.7-2.3) of children face CMA, making milk the second-most prevalent allergy among children (25.4% of children affected by food allergy) [4]. Previously thought to be a strictly children’s disease that many grow out of, surprisingly, adults face mirroring rates with an overall prevalence of 1.9% (95% CI: 1.8-2.1) [3].

The hypersensitivity reactions outlined above resemble allergies that have been definitively diagnosed (i.e., confirmed through different diagnostic techniques). This greatly differs from food intolerance. A food allergy is an abnormal immune response to any food component (e.g., protein, carbohydrate, and additives) [18]. In this paper, although type I is considered a true allergy, for the sake of simplicity and cohesiveness, we have noted type IV within this description as well. This has been demonstrated in many of the studies included in this review. Contrary to food allergies, food intolerances do not necessarily elicit immunological responses but rather produce adverse reactions through many various mechanisms (e.g., toxic, metabolic, and allergic) caused by the food or a component of the food [18,41]. With this, there typically exist different symptoms when deciphering between food allergies and food intolerances, although commonalities exist. Various studies have shown intolerance to be commonly associated with the carbohydrate component of milk (e.g., lactose) [42], whereas allergies are primarily derived from a protein component, as discussed previously (e.g., whey or casein) [18,41].

After ingestion of the allergen (i.e., cow’s milk), those with CMA face symptoms deriving from specific reactions, in particular, primarily type I and type IV reactions. By eliciting an array of various immune mediators and effectors, patients face varying symptoms, many of which mirror symptoms seen in patients with AD (e.g., urticaria, intense pruritus, and eczematous lesions) [9]. When considering the clinical implications of CMA, it is important to note that more severe reactions can occur, including anaphylaxis, although this review article focuses solely on symptoms aligning with concomitant AD [3,4]. Due to the high prevalence and potentially serious symptoms, more research is warranted on the implications of concomitant CMA and AD.

Research surrounding infant and children populations has been conducted for decades; however, studies involving adult populations are extremely scarce. More than likely, this can be attributed to the notion that CMA has a relatively good recovery rate in adult populations, with one recent study noting a total recovery of 87% at three years, 92% at five years, and 97% at both 15 and 26 years of age [24]. However, this study also notes that those diagnosed with CMA within the first year of life are at a higher risk for developing atopic dermatitis, among other atopic diseases, despite a decreasing prevalence [24]. Of the limited publications, research has been restricted to small cohorts, thus limiting the representation of the general population. Additionally, many older studies are hampered by outdated technology and methodology while providing obscure data and methods of derivation, leading to confusing and contradicting ideas and results. With these limitations, we do understand the difficult tasks involved in conducting research surrounding this topic, in particular, a lack of diagnostic testing, shortfalls in milk product standardization, and countless confounding variables, just to name a few.

In the literature, it seems that a lack of diagnostic testing exists when deriving a diagnosis of CMA [18]. Of the quickest and most cost-effective methods, skin prick and patch tests, these modalities have been shown in many studies to provide positive results without clinical symptoms present [16,18,22]. Another testing modality frequently used is the allergen-specific serum IgE test. This test determines the absence or presence of specific IgE antibodies within the serum with the goal of identifying specific allergens that generate immune responses [18]. As mentioned previously, this test also lacks diagnostic capabilities as a single test due to a lack of clinical symptoms despite deriving a positive result in some cases [18]. In addition, this test fails to include patients whose immune systems are following type IV mechanisms that involve pathways other than IgE proliferation. Although deemed nondiagnostic when used alone, Yanagida et al. demonstrated a positive correlation between IgE antibody levels (resembled by IgE-specific testing) and risk for anaphylaxis in children (mean age: 3.5 years); no such connections were made relating to skin symptoms within the studied cohort [43]. Of all the testing modalities that exist (including some not mentioned), double-blind placebo-controlled food challenge (DBPCFC) (oral food challenge (OFC)) remains the gold standard for diagnosing food allergies [18-22]. However, with required periodical visits, a long duration many times spanning months, and the requirement of conducting the allergen reintroduction within a clinical setting due to a higher risk of anaphylaxis, DBPCFC can be very inconvenient and time-consuming [18,22,43]. With the necessity for the patient to undergo various testing due to inconsistent diagnostic capabilities, it becomes quite difficult to avoid attrition in addition to other considerations such as economical.

In addition to the observed diagnostic variance, an additional variance is applicable when considering the preparation and chemical composition across milk products. As exemplified in the study of Foroutan et al. examining the micro-composition of cow’s milk, many milk products contain differing concentrations of allergy inducers, most notably the various casein proteins [6]. With increased 𝛽-casein concentrations, particularly A1 𝛽-caseins, significantly increased associated symptoms (e.g., abdominal discomfort, bloating, and borborygmus) were seen; dermatological manifestations were not included in this particular study [42]. Additionally, a lack of standardization in the milk-making process limits the applicability of the research when considering various populations across different countries and regions whose regulations vary. On the contrary, many developed countries have initiated overarching organizations that ensure consistency throughout the homogenization and pasteurization processes [44]. This not only ensures safety for the consumer but also creates a standardization when creating milk products. However, many countries, including the United States, have regulatory codes that only require certain minimums to be met rather than ranges [44]. For example, under Australia and New Zealand Food Standards Code, full-fat milk must contain at least 3.2% of fat and 3% of protein [44]. Although allowing more freedom to corporations in the production of milk products, those affected by CMA may face added symptoms when consuming certain brands.

Lastly, confounding variables remain a large concern, especially when conducting food allergy trials. Liquid milk is relatively straightforward when attempting to avoid cow milk products. However, many studies note patient difficulties when attempting to avoid milk-derived products. These include butter, bread, and dressings, along with more discreet milk-containing products such as flavored chips, medications, granola mixes, and even certain vitamin supplements. In addition to this obscurity, the under-labeling of food items has added an extra burden for consumers to avoid certain food categories, specifically, milk. The US Food and Drug Administration (FDA) has named milk a “major recall source” due to under-labeling [1]. To combat this, the passing of the Food Allergen Labeling and Consumer Protection Act (FALCPA) of 2004 has placed stricter guidelines for food companies and has increased enforcement under such protocols. This law requires that major allergens (milk, eggs, fish, shellfish, tree nuts, peanuts, wheat, and soybeans) must be shown explicitly, either within the ingredient list (e.g., “whey (milk)”) or in a separate section following the ingredients list (e.g., “contains milk”) [1]. With this in place, there exists less consumer confusion when attempting to exclude certain food groups within the diet, such as needed in DBPCFC tests for delineating food allergy manifestations.

Cow’s milk allergy remains among the top allergies affecting the United States today, with numbers steadily increasing each year [3,4]. In addition, eczema remains the most common inflammatory skin disease in the United States [13]. Considering the severity, prevalence, and quality of life implications set forth by such diseases, more research is warranted within the field. By outlining the underlying interconnected relationships between food allergy and atopic dermatitis, our goal throughout this paper was to identify the limited major studies that exist while highlighting the downfalls seen within this realm of research. Although highlighting parallels between symptomology and pathophysiology of CMA and AD, substantial confusion still exists when studying these relationships. Limited by a lack of diagnostic testing, hampered standardization, and consumer confusion, we understand research within this field is quite difficult.

## Conclusions

Many populations face CMA with the added burden of the appearance or worsening of AD when consuming milk products. In the United States, specifically, research on this topic is greatly scarce, especially when considering the adolescent and adult populations. However, due to the prevalence and possible severity of symptoms, it is imperative to understand the underlying immune mechanisms involved in such reactions. Although the aforementioned difficulties exist in conducting research on this topic, we believe that more studies are warranted to further understand the immunological relationships between cow’s milk allergy manifesting as atopic dermatitis. For future studies, we hope for more well-conducted studies characterized by larger cohorts, standardized methodologies, and clearly outlined objectives and results. It is through advanced research that we hope to further understand the underlying immune mechanisms involved. With this, future treatment can become more targeted in combating the complex manifestations of cow’s milk allergy manifesting as atopic dermatitis.
